# Self-Healing Performance of Multifunctional Polymeric Smart Coatings

**DOI:** 10.3390/polym11091519

**Published:** 2019-09-18

**Authors:** Sehrish Habib, Adnan Khan, Muddasir Nawaz, Mostafa Hussein Ramadan Sliem, Rana Abdul Shakoor, Ramazan Kahraman, Aboubakr M. Abdullah, Atef Zekri

**Affiliations:** 1 Center of Advanced Materials (CAM), Qatar University, 2713 Doha, Qatar; sehrish.habib88@gmail.com (S.H.); ak1704740@qu.edu.qa (A.K.); m.nawaz@qu.edu.qa (M.N.); mostafa@qu.edu.qa (M.H.R.S.); bakr@qu.edu.qa (A.M.A.); 2 Department of Chemical Engineering, Qatar University, 2713 Doha, Qatar; ramazank@qu.edu.qa; 3 Qatar Energy and Environment Research Institute, Hamad Bin Khalifa University, Qatar Foundation, 34110 Doha, Qatar; azekri@hbku.edu.qa

**Keywords:** polymeric, nanocomposite, halloysite nanotubes, inhibitor, self-healing, corrosion

## Abstract

Multifunctional nanocomposite coatings were synthesized by reinforcing a polymeric matrix with halloysite nanotubes (HNTs) loaded with corrosion inhibitor (NaNO_3_) and urea formaldehyde microcapsules (UFMCs) encapsulated with a self-healing agent (linseed oil (LO)). The developed polymeric nanocomposite coatings were applied on the polished mild steel substrate using the doctor’s blade technique. The structural (FTIR, XPS) and thermogravimetric (TGA) analyses reveal the loading of HNTs with NaNO_3_ and encapsulation of UFMCs with linseed oil. It was observed that self-release of the inhibitor from HNTs in response to pH change was a time dependent process. Nanocomposite coatings demonstrate decent self-healing effects in response to the external controlled mechanical damage. Electrochemical impedance spectroscopic analysis (EIS) indicates promising anticorrosive performance of novel nanocomposite coatings. Observed corrosion resistance of the developed smart coatings may be attributed to the efficient release of inhibitor and self-healing agent in response to the external stimuli. Polymeric nanocomposite coatings modified with multifunctional species may offer suitable corrosion protection of steel in the oil and gas industry.

## 1. Introduction

In the oil and gas industry, corrosion is considered to be the most damaging phenomenon which causes major changes in materials resulting in massive economic loss, safety threats, and unfortunate accidents if not addressed in a timely manner [1,2]. Various methods have been developed to prevent materials from corroding, including cathodic protection, coatings, and use of corrosion inhibitors [3,4,5]. The protection of materials through applying suitable coatings has been considered to be among the most effective methods for many years [6]. However, once the coating is damaged or detached, the system cannot stop the corrosion process [7]. In order to improve the performance of coatings to meet the industrial requirements, coating systems known as ‘smart coatings’ have been developed with attractive multifunctional characteristics such as self-healing and self-inhibition [8,9,10]. Any external stimuli, such as a change in pH, metal ions, and/or electrochemical interactions, causes triggering of multifunctional responses [11].

Corrosion rate has been effectively concealed with the addition of appropriate inhibitors. Earlier chromate based coatings were used to protect the metals against corrosion but those were found to be carcinogenic which eventually led to their prohibition [5]. The inhibitors need to be environment friendly, less toxic, and less polluting [12,13]. Organic corrosion inhibitors such as benzotriazole (BTA) [14], thioaldehydes [15], and papaverine [16] and inorganic corrosion inhibitors composed of phophates, sulphates, nitrites, and nitrates are widely used these days providing effective inhibition in response to specific stimuli [17]. However, previous studies have reported that direct addition of any inhibitor into a coating system is not adequate because of reasons like (i) its reaction with coating materials and (ii) its solubility in coatings resulting in low concentration of inhibitor and thus poor barrier properties [18,19]. An advanced solution to avoid any interaction of the inhibitor and the coating matrix is the encapsulation or loading of the inhibitor into nanocontainers [7,20]. Nano containers can insulate the loaded/encapsulated inhibitor, thus avoiding leakage and provide its controlled release when and where required [21].

The multifunctional coatings with inhibitors and self-healing agents are considered to be promising. The concept of corrosion inhibition with self-healing helps to heal cracks/damages automatically without an external intrusion [22]. The encapsulation of self-healing species that are sensitive to any external stimuli (pH, moisture, light, or crack propagation) is attractive to introduce functionalities that can heal the damage. Various coating systems with self-healing agents have been studied [23]. One of the most studied smart coating systems is the one composed of urea formaldehyde microcapsules (UFMC) encapsulated with various inhibitors and self-healing agents reinforced into polymeric matrix [24].

Various nanocontainers have been introduced including polymer containers [25], halloysite [26], ion exchange organic resins [27], nanocontainer with a polyelectrolyte shell [28], layered double hydroxide (LDH) conductive polymer matrix [29], montmorillonite [30], and mesoporous inorganic materials [31]. Haddadi et al. [32] successfully synthesized a carbon hollow sphere using the silica templating method and encapsulated 2-mercaptobenzimidazole (MBI) inhibitor. Karekar et al. [33] studied the release of benzotriazole (BTA) from a layer by layer nanocontainer based on zinc molybadate. Ubaid et al. [34] synthesized titania nanotubes (TNTs) using the hydrothermal method and encapsulated with dodecyl amine (DOC) as the corrosion inhibitor to study the effect of concentration of DOC loaded TNTs on corrosion protection of steel. Xing el al [1] successfully loaded Na_2_MoO_4_ into halloysite nanotubes (HNTs) and encapsulated them with a BTA-Cu complex to guard carbon steel against corrosion. Shchukina et al. [21] carried out a comparative study of 8-hydroxyquinoline loaded into HNTs and mesoporous silica on corrosion protection of polyepoxy powder coating and successfully inhibited the formation of pitting corrosion. Falcon et al. [35] produced SiO_2_ nanocontainers using the layer by layer technique to encapsulate DOC for corrosion protection of steel. However, synthesis of these nanocontainers is not cost friendly limiting their application. An alternative way is to use natural occurring biocompatible and environmental friendly nanocontainers such as halloysite nanotubes (HNTs) [18,36,37,38]. Halloysite is an alumino-silicate nanoclay (Al_2_Si_2_O_5_(OH)_4_·2(H_2_O)) which exhibits a cylindrical arrangement with exceptional hollow core structure or successions of voids with a diameter range of 16–50 nm. This unique structure is suitable for trapping active agents within the walls of cylinders [39] and thus making HNTs a promising delivery system to develop smart coatings.

**Figure 1:**
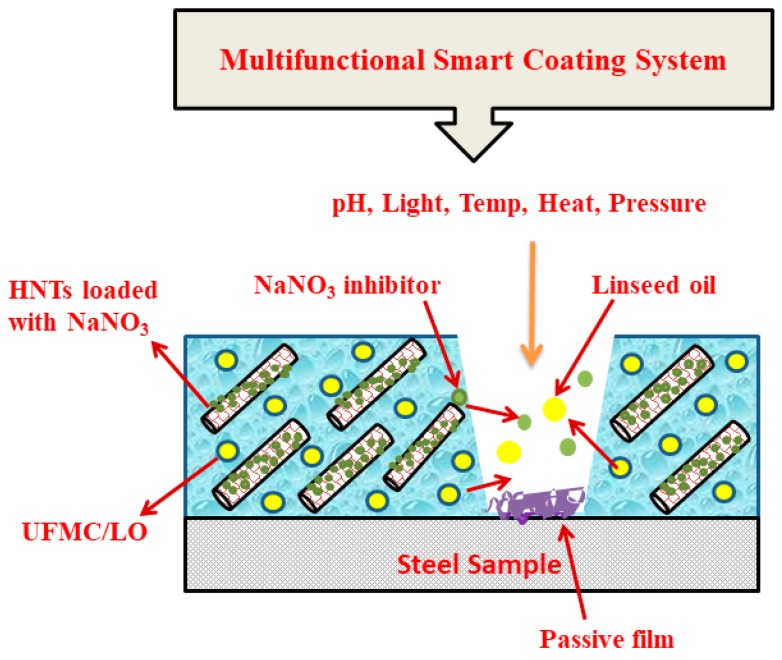
Schematic representation of multifunctional nanocomposite coating concept.

In this work we studied the self-healing performance of novel smart nanocomposite coatings composed of HNTs loaded with NaNO_3_ (corrosion inhibitor) and urea formaldehyde microcapsules encapsulated with linseed oil (self-healing agent). The coatings were prepared by reinforcing a polymeric matrix with HNTs loaded with NaNO_3_ and UFMCs encapsulated with linseed oil named as UFMC/LO. The structural, morphological, thermal, chemical, and electrochemical characterizations of the prepared nanocomposite coatings were conducted to elucidate the beneficial role HNTs, the self-healing agent, and the corrosion inhibitor in multifunctional nanocomposite coatings to combat corrosion. The results reveal that the developed polymeric nanocomposite coatings demonstrate decent anticorrosion properties due to appropriate selection of nanocontainers and compatible self-healing agent and inhibitor. The improved performance may also be attributed to the competent coating system that ensures efficient release of the self-healing agent and the inhibitor in response to external stimuli. Self-healing ability and corrosion inhibition effects of the polymeric smart coatings have been combined together in a single layer as schematically shown in Figure 1.

## 2. Experimental

### 2.1. Materials and Chemicals

Halloysite nanotubes (HNTs) were used as nano containers, purchased from Sigma Aldrich, Darmstadt, Germany. Sodium nitrate (NaNO_3_) was used as corrosion inhibitor, also procured from Sigma Aldrich, Darmstadt, Germany. The other chemicals and materials used in the study are linseed oil, epoxy resin (815 °C) and its curing agent, hydrochloric acid, sodium hydroxide pallets, urea, formaldehyde, ammonium chloride (NH_4_Cl), resorcinol, and polyvinyl alcohol (PVA) which were also obtained from Sigma Aldrich, Darmstadt, Germany. Carbon steel (30 × 30 × 1.0 mm^3^) used as substrates were purchased from a local source. The substrates were cleaned and polished using silicon carbide (SiC) abrasive papers purchased from Hebei Yineng Pipeline Group Co., Ltd, China. The prepared substrates were thoroughly rinsed with water and finally cleaned with ethanol before applying coatings.

### 2.2. Loading of Corrosion Inhibitor

Sodium nitrate (NaNO_3_), the corrosion inhibitor, was loaded into halloysite nanotubes (HNTs) following the vacuum cycling method as reported by Price et al. [39]. Firstly, NaNO_3_ (6.0 g) was dissolved into distilled water (50.0 mL) as purchased HNTs (3.0 g) were added into this solution and put into a sonicator to form a suspension. The suspension was then transferred into a vacuum chamber having a pressure of 10^−3^ mbar. Because of the maintained vacuum, air is removed from the HNTs and replaced by NaNO_3_. This vacuum condition was maintained for some appropriate time to ensure the loading process and then was cycled back to atmospheric pressure. This process was repeated many times and suspension was placed in this sealed condition for 24 h. The suspension was then centrifuged at 5000 rpm for 30 min to collect the separated product. The supernatant was removed and the product was dried in an oven at 60 °C to remove moisture from it. The dried powder was collected and saved for further characterization and coating.

### 2.3. Synthesis of Urea Formaldehyde Microcapsules (UFMCs) and Their Encapsulation 

Urea formaldehyde microcapsules (UFMCs) were synthesized by the in-situ polymerization technique [40]. During this process, 290.0 mL of deionized water and 10.0 mL (5.0 wt% of aqueous solution) of PVA were mixed in a beaker at room temperature. During stirring, 5.0 g of urea, 0.5 g of NH_4_Cl, and 0.5 g resorcinol were dissolved in the solution by adjusting the pH to 3.5. After adding 1–2 drops of octanol as an antifoaming agent, 30.0 mL of linseed oil was also added slowly into the solution and allowed to stabilize for 10 minutes. After that, 11.6 mL (37 wt% aqueous solution) of formaldehyde was added by constant stirring at 900 rpm and the reaction temperature was set at 55 °C for 4 h. After cooling down to room temperature, the microcapsules were then washed with distilled water and then with xylene. The microcapsules were then filtered and dried under vacuum.

### 2.4. Characterization of Loaded Nanotubes and Urea Formaldehyde Microcapsules

The morphology and elemental composition of the synthesized UFMCs and HNTs loaded with corrosion inhibitor was studied by a field emission scanning electron microscope (FE–SEM–Nova Nano-450, FEI, New York, NY, USA) coupled with Energy-dispersive X-ray spectroscopy (EDX) tool. A transmission electron microscope (TEM TALOS F200X, FEI, New York, NY, USA) was used to study the microstructure of halloysite nanotubes. The structural and phase analysis was performed through X-ray diffraction analysis (PANanalytical, Empyrean, Royston, United Kingdom) with a scanning rate of 2°/min and scanning angle ranging between 10° ≤ 2θ ≤ 90°.

Fourier transform infrared (FTIR) spectra were recorded using a FTIR Frontier (PerkinElmer, Waltham, MA, USA) spectrometer in the range of 4000–500 cm^−1^ to study the presence of functional groups of HNTs loaded with the inhibitor and UFMCs encapsulated with linseed oil.

In order to have deep insight into the synthesized smart containers and to confirm the loading/encapsulation of the inhibitor and the self-healing agent, HNTs and UFMCs were also characterized by X-ray photoelectron spectroscopy (XPS) (AXIX Ultra DLD, Kratos, UK) employing a monochromatic X-ray source (Al Kα source). XPS survey spectra were recorded in the binding energy range of 0 to 800 eV and the binding energy of C 1s (284.6 eV) was used as reference. Thermal stability studies of as received HNTs, HNTs loaded with the inhibitor, and UFMCs were conducted using Thermogravimetric analysis (TGA) analyzer pyris 4000 (PerkinElmer) ranging from 30 to 600 °C at the heating rate of 10 °C/min. Size distribution of the prepared microcapsules was determined using a particle size analyzer (Malvern, Master Sizer 2000, Panalytical, USA). To study the release of the inhibitor from HNTs at different pH values, UV-Vis spectroscopic analysis (LAMBDA 650 UV/Vis Spectrophotometer, PerkinElmer) was conducted. For this purpose, a small amount (0.5 g) of loaded product was added into 5.0 mL of 0.1 M NaCl solution to form suspension. pH of the solution was adjusted at 2, 5, 7, 9, and 11 by adding suitable amounts of HCl or NaOH and the released amount of NaNO_3_ was plotted as a function of time for analysis.

### 2.5. Synthesis of Smart Coatings

HNTs at 5.0 wt% loaded with NaNO_3_ (corrosion inhibitor) and 5.0 wt% UFMCs encapsulated with linseed oil (self-healing agent) microcapsules were added into the epoxy resin (5.0 g). After that, curing agent (1.25 g) was added and the mixture was stirred for 5 min. In order to make a uniform dispersion, the mixture was continuously stirred for 10 min. This coating mixture was then applied on steel substrates using the doctor’s blade technique. The samples were left for curing for 2 weeks until the coating was completely dried and hardened with a final thickness of 150 µm.

### 2.6. Characterization of Smart Coatings

FE–SEM was used to study the dispersal of HNTs loaded with NaNO_3_ and UFMCs encapsulated with linseed oil in the epoxy matrix. EDX was employed to study the elemental composition of the developed coatings. The structural analysis of coatings was done by FTIR spectroscopy. Thermal stability of the synthesized coatings was evaluated by TGA analysis. The self-healing capability of the synthesized coating was analyzed through controlled damage of the coating. For this purpose, the coated samples were artificially scratched and the healing process was observed through SEM.

**Figure 2:**
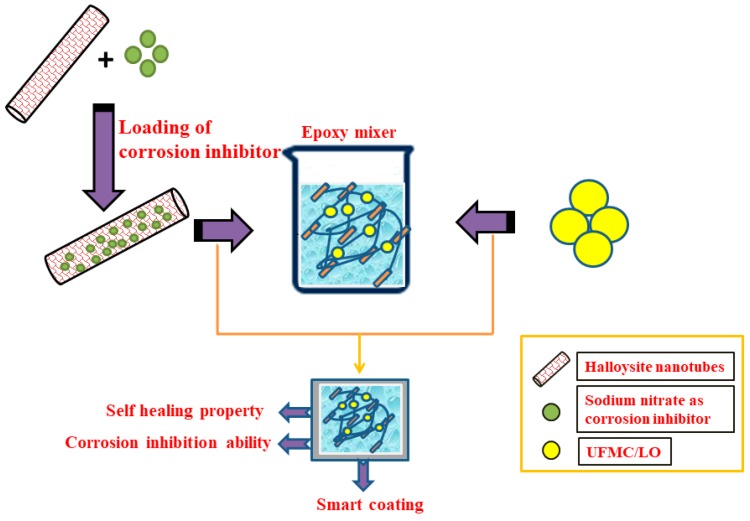
Schematic representation of complete experimental procedure adopted.

The anticorrosion properties and self-healing abilities of the synthesized coatings in 3.5 wt% NaCl solution were investigated using the electrochemical impedance spectroscopy (EIS) technique. For this purpose, the coatings were subjected to a controlled damage and then put into EIS testing. The electrochemical measurements were carried out using a Gamry 3000 (30K BOOSTER Potentiostat/Galvanstate/ZRA, USA) having a three electrode system. In this study, the coated sample was used as the working electrode whereas the graphite rod and Ag/AgCl were used as the counter and the reference electrodes, respectively. EIS measurements were commenced after attaining a steady value for the open circuit potential. The frequency range for the EIS experiment was within 0.1–100 kHz from higher to lower value and the root mean square (RMS) signal was 10.0 mV. The measured EIS data were analyzed by Gamry E-Chem 3000 software and fitting parameters were determined by the suitable equivalent circuits. Figure 2 schematically represents the complete experimental methodology adopted in the current study.

## 3. Results and Discussion

### 3.1. Morphological and Structural Analysis

**Figure 3:**
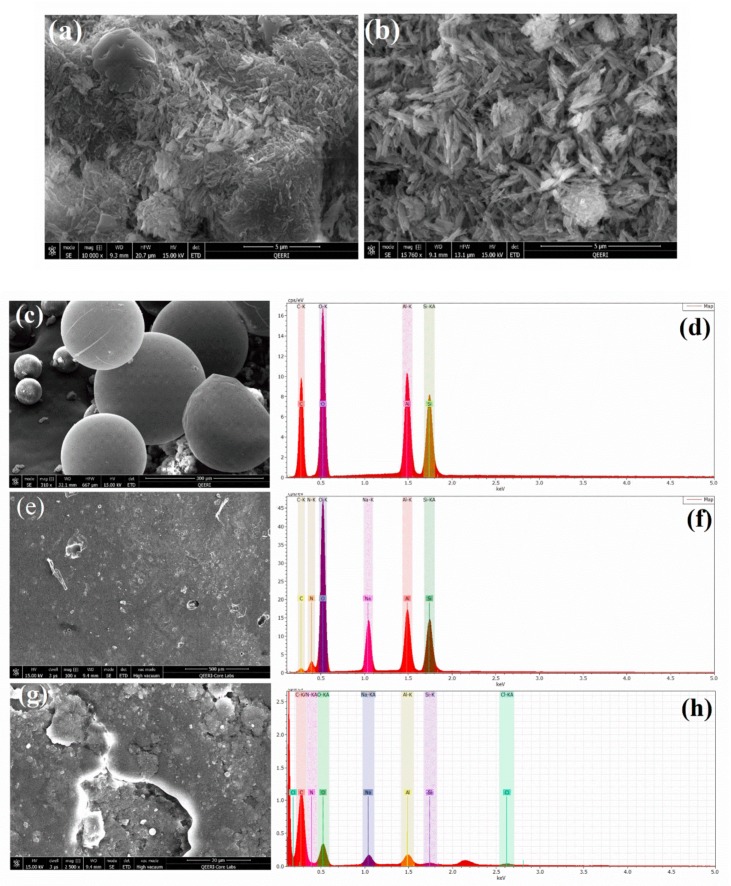
Field emission scanning electron microscope (FE–SEM) analysis of: (**a**) halloysite nanotubes (HNTs), (**b**) HNTs loaded with NaNO_3_, (**c**) urea formaldehyde microcapsules/linseed oil (UFMC/LO), and (**e**,**g**) nanocomposite coatings; and EDX analysis of: (**d**) HNTs, (**f**) HNTs loaded with NaNO_3_, and (**h**) nanocomposite coatings.

Figure 3a–c shows the scanning electron microscopic (SEM) analysis of HNTs, loaded HNTs, and UFMCs. In Figure 3a, HNTs display a elongated hollow rod like structure with accretion of tubes. Figure 3b shows that HNTs loaded with the inhibitor reveal a smooth surface similar to pristine HNT confirming the absence of adsorbed NaNO_3_ on the surface of HNTs. A comparison of EDX analysis of HNTs (Figure 3d) and HNTs loaded with NaNO_3_ (Figure 3e) indicates that HNTs loaded with NaNO_3_ are comprised of Na, N, and O confirming the presence of NaNO_3_ in HNTs. The SEM images of UFMCs encapsulated with linseed oil are shown in Figure 3c. A denser and more diffused structure is achieved. A spherical morphology of UFMCs is achieved without any cracks. There is some variation in the sizes of microcapsules depending on the stirring speed employed during the synthesis process. It is reported that high stirring speed usually leads to finer spherical morphology [41]. The rough outer surface of the microcapsules shown in SEM develops better bonding to the coating matrix. Figure 3e,g shows the SEM images for nanocomposite coating.

**Figure 4:**
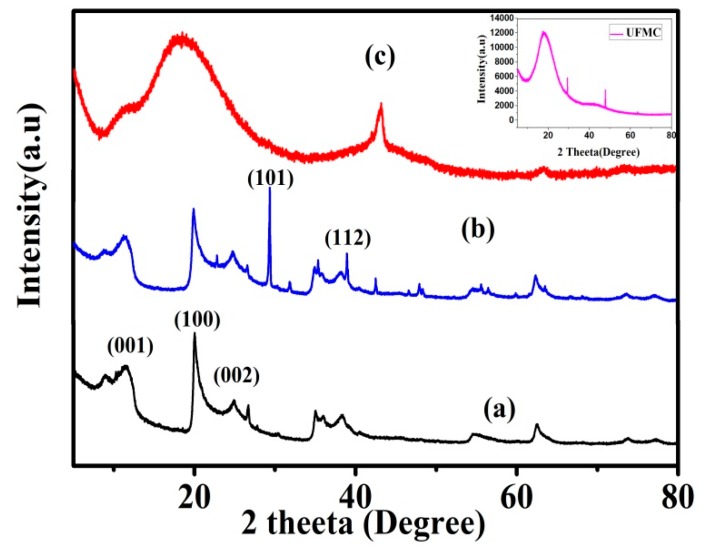
XRD analysis: (**a**) HNTs, (**b**) HNTs loaded with NaNO_3_, and (**c**) Nanocomposite coatings. Inset shows the XRD of UFMCs.

Figure 4a,b shows the XRD spectra of HNTs and HNTs loaded with NaNO_3_. The sharpness of peaks and the absence of any extra peaks confirm the formation of high purity crystalline HNTs. HNTs show diffraction peaks and designated crystal planes at 2Ө = 11.7° (001), 20.0° (100), and 25.003° (002) [1]. After the loading of NaNO_3_ into HNTs, the XRD pattern shows characteristic peaks of HNTs with some new peaks at 2Ө = 29.6°, 32.16°, 61.6° corresponding to (110), (002), and (220) planes of NaNO_3_ (ICSD:174034, ICDD:98-017-4034) confirming the loading of the inhibitor into HNTs. A typical XRD pattern of the epoxy coating is attained by incorporation of HNTs loaded with NaNO_3_ and UFMCs encapsulated with linseed oil into the epoxy matrix to form nanocomposite coatings as shown in Figure 4c. XRD spectrum of the encapsulated UFMCs (inset) is also included in the figure showing the amorphous behavior of the microcapsules and the diffraction peak at 17.5° accounting for the urea formaldehyde as a shell material encapsulating linseed oil as reported in the literature [28].

### 3.2. FTIR Analysis of HNTs, UFMCs, and the Nanocomposite Coatings

**Figure 5:**
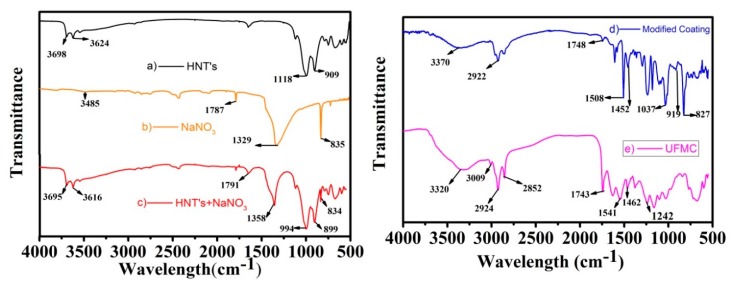
Fourier transform infrared (FTIR) spectra of (**a**) HNTs, (**b**) NaNO_3_, (**c**) HNTs loaded with NaNO_3_, (**d**) nanocomposite coatings, and (**e**) UFMCs.

The FTIR spectra of HNTs, NaNO_3_, HNTs loaded with NaNO_3_, encapsulated UFMCs, and the nanocomposite coatings are presented in Figure 5a–e. The FTIR spectrum of pristine HNTs (Figure 5a) confirms the presence of Al_2_O-H stretching absorption band at 3698 and 3624 cm^−1^, two Al atoms are linked to each OH in plane Si–O–Si stretching at 1118 cm^−1^ and Al_2_O-H deformation band at 909 cm^−1^ [42]. A comparison of three spectra as shown in Figure 5a–c displays that NaNO_3_ loaded in HNTs showed N=O stretching band at 1791 cm^−1^, and NO_3_ asymmetric and symmetric stretching bands at 1358 and 899 cm^−1^, respectively [43]. The shifts in the peaks of NaNO_3_ from 1787 to 1791 cm^−1^, 1329 to 1358 cm^−1^, and 835 to 899 cm^−1^ can be due to the hydrogen bonding involved in loading of NaNO_3_ into HNTs. The FTIR spectra presented in Figure 5a–c confirms the loading of NaNO_3_ into HNTs.

FTIR spectrum of encapsulated UFMCs is shown in Figure 5e. The spectrum shows characteristic peaks of both urea formaldehyde and linseed oil. The broad absorption band at 3320 cm^−1^ shows overlapping of the N–H bonds and O–H bond and can be assigned to urea–formaldehyde. The small sharp peak at 3090 cm^−1^ represents the C–H bands, while peaks at 2924 cm^−1^ and 2852 cm^−1^ show the presence of O–H and C–H stretching band. There is another sharp peak at 1743 cm^−1^ representing the carbonyl C=O bands. These bands are evidence for the presence of linseed oil. There is also a new peak at 1541 cm^−1^ representing the N–H band and its presence indicates the existence of urea–formaldehyde. The peak at 1462 cm^−1^ also represents a C–H band with a different vibration and the peak at 1242 cm^−1^ corresponds to the C–N band. The C–H and C–N vibrations are present in both UFMCs and pure linseed oil [44]. The presence of corresponding distinctive absorption bands of N–H at 1541 cm^−1^ (urea formaldehyde), C=O at 1743 cm^−1^ (linseed oil), and C–N at 1242 cm^−1^ (linseed oil) in the UFMCs confirm the encapsulation of linseed oil.

In case of nanocomposite coatings (Figure 5d), the peaks at 827, 919, 1037, 1452, 1508, and 2922 cm^−1^ are assigned to oxirane, stretching of oxirane, stretching of C–O–C of ether, –CH_3_ deformation, and C–C aromatic, respectively, which are in accordance with the previous results [45,46,47]. The FTIR analysis confirms the successful loading of NaNO_3_ into HNTs and encapsulation of linseed oil in UFMCs leading to the development of nanocomposite coatings upon their reinforcement into the epoxy matrix.

### 3.3. XPS Analysis

**Figure 6:**
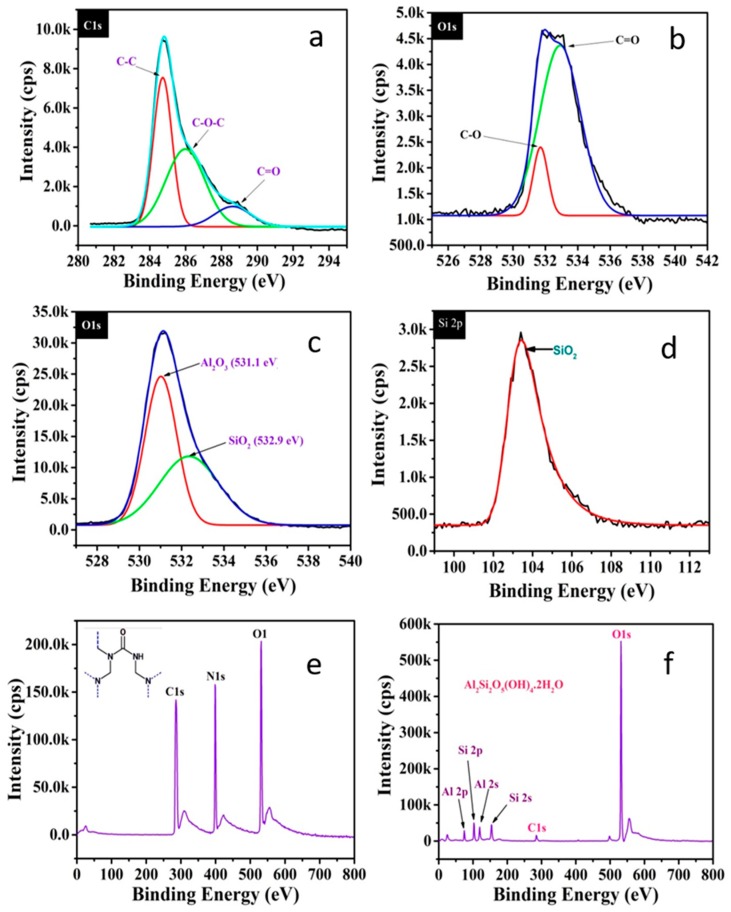
XPS spectra of synthesized containers; (**a**,**b**) C1s and O1s spectrum of UFMCs, (**c**,**d**) O1s and Si2p spectrum of HNTs, and (**e**,**f**) survey spectrum of UFMCs and HNTs.

The synthesized containers were subjected to XPS analysis and the XPS spectra were recorded in the binding energy range of 0 to 800 eV as shown in Figure 6a–f. Carbon, oxygen, and nitrogen were the main elements detected in the surface of UFMCs while oxygen, aluminum, and silica were identified in HNTs, as expected. Figure 6a,b shows the XPS spectra of carbon and oxygen in UFMCs while Figure 6c,d represents the oxygen and silicon spectra of HNTs. For the UFMCs, carbon peaks at 284.8 eV, 286.8 eV, and 288.4 eV represent the bonding energy of C–C, C–O–C, and C=O, respectively. The oxygen peaks of UFMCs indicate the bonding energy of C–O and C=O. In the case of HNTs, the oxygen of the HNTs showed distinct fitting peaks (at 531.1 eV and 532.9 eV) which reflect the bonding energy of Al_2_O_3_ and SiO_2_ while that of the silicon XPS spectra shows only the bonding energy of SiO_2_. The mass concentration of oxygen and silicon present in the HNTs were about 69.39 and 25.17%, respectively. The results also confirm the presence of ~2–4% carbon which is considered as the organic impurities in the sample. Figure 6e,f show the survey spectra of UFMCs and HNTs with the entire elemental peaks present in their structure.

The XPS study confirms that phase pure containers have been synthesized without any impurities and the results are in agreement with the previously published reports [48,49]. It is also confirmed that the inhibitor (NaNO_3_) and the self-healing agent (linseed oil) are present inside the core of the containers rather than sticking on the surface with absence of any related elements to NaNO_3_ and linseed oil.

### 3.4. Particle Size Analysis of the Urea Formaldehyde Microcapsules

**Figure 7:**
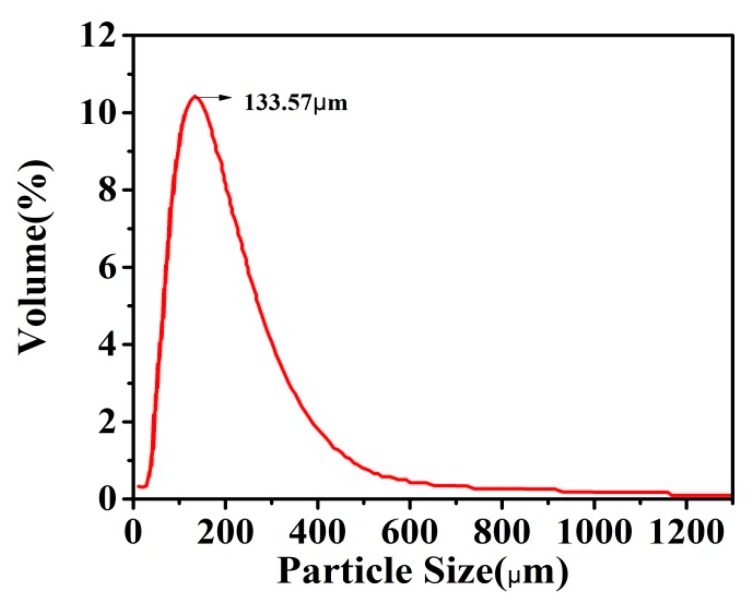
Particle size analysis of the synthesized UFMCs.

Figure 7 and Table 1 show the particle size of the UFMCs. The sizes of the microcapsules are in the range of 0.01–2000 μm. The majority of the particles resides in the range of 125–250 μm, while only 0.13% are in the range of 1000–2000 μm. This analysis also shows that the average size of the UFMCs is ~133.57 μm. Particle size depends on the stirring speed; the higher the stirring speed, the smaller and finer the particle size [41].

### 3.5. Thermal Stability of the Smart Containers and Nanocomposite Coatings

**Figure 8:**
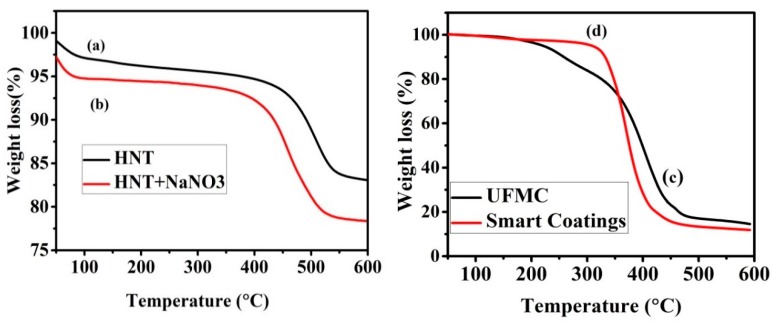
Thermogravimetric (TGA) curves: (**a**) pristine HNTs, (**b**) HNTs loaded with NaNO_3_, (**c**) encapsulated UFMCs, and (**d**) nanocomposite coatings.

Thermal stabilities of HNTs, HNTs loaded with NaNO_3_, UFMCs, and the smart coating were studied by thermogravimetric analysis. It is observed from Figure 8a that HNTs show good thermal stability up to 400 °C without any significant weight loss. The initial weight loss (at 100 °C) was probably due to evaporation of moisture present in the HNTs. In the second stage, there was no prominent weight loss and weight almost remained constant until 380 °C. However, with further increases in temperature (above 500 °C), weight loss of ~13 to 15% was noticed which can be ascribed to the loss of interlayer water due to the dehydration process [50]. In the case of HNTs loaded with NaNO_3_ (Figure 8b), similarly, three stages in the TGA curve were observed. In the first stage, about 2% weight loss was noticed which may be due to the loss of moisture content in HNTs. In the second stage, there was no prominent weight loss and weight almost remained constant until 380 °C similar to pristine HNTs. However, further increases in temperature resulted in a significant weight loss unlike the pristine HNTs (Figure 8a) which is mainly due to the decomposition of NaNO_3_ into NaNO_2_ and oxygen gas. This behavior was expected due to the boiling point of NaNO_3_ (380 °C). Further heating leads to decomposition of NaNO_2_ into sodium oxide and nitrogen oxide. Based on the weight loss during the TGA analysis, we can assume that ~5.0 wt% of NaNO_3_ was loaded into the HNTs.

Figure 8c presents the TGA of UFMCs. There was gradual weight loss with increasing temperature up to 500 °C. Initially (up to 200 °C), the weight loss was relatively slow which may be associated to the removal of absorbed moisture content in the UFMCs. In the next stage (200 to 400 °C), the UFMCs showed rapid weight loss which can be associated with the decomposition of encapsulated linseed oil into UFMCs and UF resin. The degraded curve at 230 °C is attributed to decomposition of UF [40]. The boiling point of linseed oil is about 175 °C. These results further confirm that microcapsules contain both linseed oil and UF. The thermal stability of the nanocomposite coatings was also evaluated as shown in Figure 7d. The synthesized coatings did not show substantial weight loss until about 350 °C and a weight loss of about 4 to 5% was noticed. However, after 350 °C, there was considerable weight loss (80–85%) when the temperature was increased from 350 to 500 °C. This weight loss can be ascribed to the breakdown of long chains in the epoxy resin bond. After 500 °C, the weight loss was because of other additives in the coating. These results are in agreement with the previous studies [51,52].

### 3.6. Self-Release of Inhibitor from Nanocontainers

**Figure 9:**
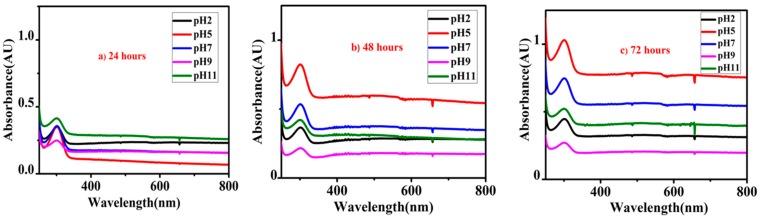
UV-Vis spectra of HNTs loaded with inhibitor immersed in 0.1M NaCl solution at various pH levels for different immersion time intervals, (**a**) 24 h, (**b**) 48 h, and (**c**) 72 h.

Figure 9a–c shows the UV-Vis spectra of HNTs loaded with the inhibitor. For this purpose, HNTs loaded with the inhibitor were immersed into 0.1 M NaCl solution at five different pH values (2, 5, 7, 9, and 11) and UV-Vis spectra were recorded at different intervals of immersion time (24, 48, and 72 h). For all the pH values, an absorption peak at about 300.0 nm was present in the UV-Vis spectra which is the characteristic absorption peak of NaNO_3_ due to the p*← n transition [53,54]. After 24 h of immersion time (Figure 9a), a small release of inhibitor was observed at all pH values owing to the small intensity of the absorption peak. However, as the immersion time was increased to 48 h as shown in Figure 9b, the absorption peak intensity also increased for all pH values indicating higher amounts of release of inhibitor into the solution. A significant release of the inhibitor was noticed with a further increase in immersion time (72 h) for all pH values as shown in Figure 9c. This analysis suggests that release of the inhibitor is sensitive to immersion time which triggers the release of the inhibitor into the solution. Even though the inhibitor can be effective in various corrosive environments due to its release in all pH values, release of the inhibitor is more efficient at pH 5 and 7 suggesting its application in the mild acidic to neutral environment. The release of the inhibitor increases with the increase in the immersion time providing a confidence that it may provide an efficient self-healing effect.

### 3.7. Self-Healing Performance of Nanocomposite Coatings

**Figure 10:**
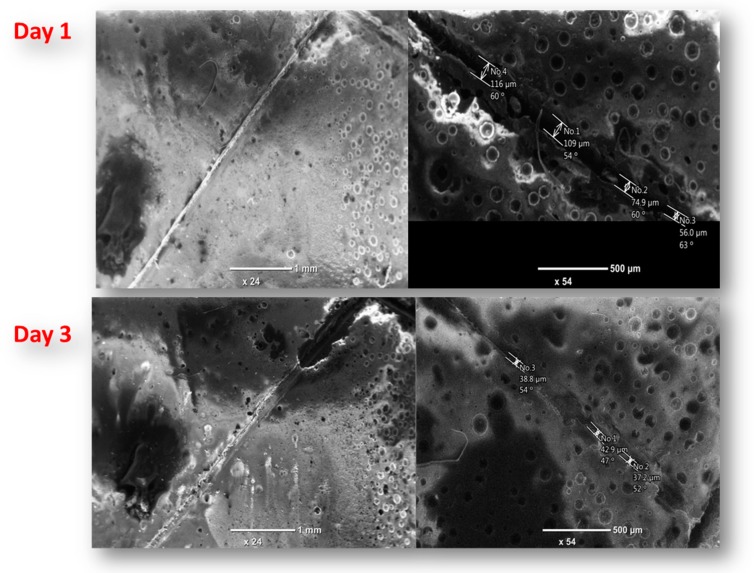
SEM images of the scratched samples after different time intervals.

The purpose of acquiring self-healing coatings is to attain good healing performance when cracks or scratches occur on the coatings. In order to achieve this objective, self-healing performance of the developed nanocomposite coatings was evaluated. During this process, the coatings were subjected to controlled damage. In order to study the self-healing performance of the developed nanocomposite coatings, SEM imaging of the damaged nanocomposite coatings was taken at different time intervals, as presented in Figure 10. There happened to be noticeable self-healing of the nanocomposite coatings with increasing time interval. The width of the crack at day 1 (upon scratching) is 109.0 µm which is reduced to 43.0 µm within 72 h (day 3) demonstrating self-healing efficiency of about 60%. The possible self-healing mechanism may be explained as below.

In response to the mechanical damage, the self-healing agent (linseed oil) is released into the crack (damaged area) from the embedded encapsulated UFMCs and fills the crack, providing a self-healing effect. The released linseed oil helps in self-healing of scratched coating and creates the passive layer to prevent direct contact of metal from the harsh environment. During this process, linseed oil polymerizes in air and a solid film is formed on the crack surface as linseed oil is cross linked to cover the crack on the coating. The new layer formed on the crack provides a considerable barrier from water and oxygen and thus provides improved corrosion resistance. The presence of a considerable amount of saturated fatty acids in linseed oil containing carbon double bond is responsible for this cross linking which auto oxides when exposed to air [24]. Furthermore, initiation of corrosion at the damaged area causes the change in localized pH that helps to trigger the release of NaNO_3_ from HNTs. It is because of the fact that the release of NaNO_3_ depends on the localized change in pH and its release helps to inhibit the further corrosion. The improvement in corrosion protection and stability of developed nanocomposite coatings can be attributed to two factors that include (i) self-healing from the cross linking of linseed oil and (ii) inhibition effect due to the release of NaNO_3._ The self-healing ability of the developed nanocomposite coatings is proven with experimental evidence which encourages the possible application of this novel coating system in many types of corrosive environments.

### 3.8. Corrosion Behavior Evaluation

**Figure 11:**
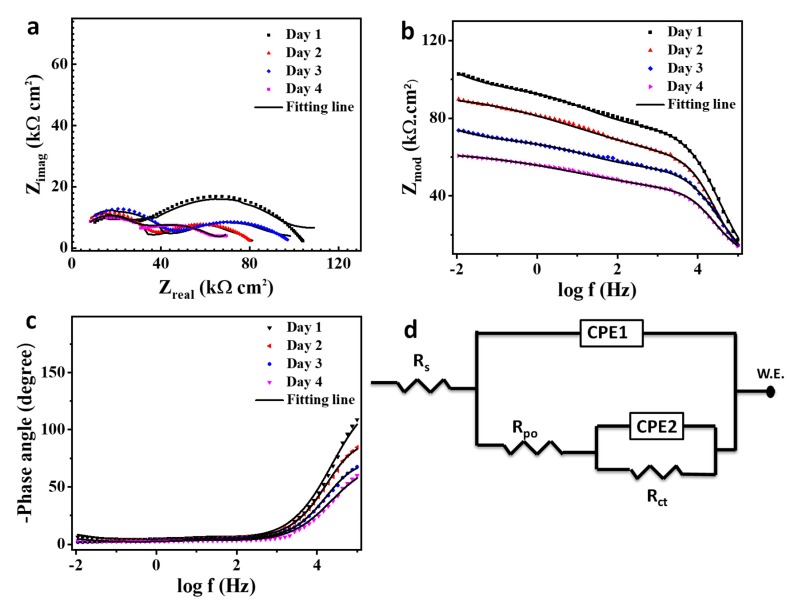
Electrochemical impedance curves for scratched pure coating immersed in 3.5 wt% NaCl for different exposure times: (**a**) Nyquist plot, (**b**) bode plot, (**c**) phase angle plot, and (**d**) the applicable equivalent circuit for the data fitting.

Figure 11 shows the EIS spectra for a scratched unmodified coating specimen after immersion in 3.5 wt% NaCl solution for different exposure times. Figure 11a–c presents the Nyquist, bode, and phase angle plot, respectively. Figure 11d presents the equivalent circuit for the EIS data analysis as the solution resistance (R_s_), pore resistance (R_po_), and charge transfer resistance (R_ct_), and constant phase elements (CPE1, CPE2) refer to the obtained electrochemical parameters from the data fitting as presented in Table 2. 

The Nyquist plot gives a typical figure of a defective coating in aqueous media with two noticeable peaks with a coating and corrosion representative response as seen in Figure 11a [55]. Corrosion resistance decreases continuously with increasing time of immersion as R_1_ looses 25% of its value after 4 days as seen in Table 2, attributed to the corrosion activity on the metal surface. The pore resistance gives a fluctuation attitude resulting from the deterioration behavior of the pure coating itself [56]. In bode and phase angle plots, two capacitive loops were observed. These loops decrease at high and low frequencies with immersion time [57]. Additionally, the magnitude of the impedance modulus is directly proportional to the capacitive loop as shown in Table 2, and the impedance value would follow the following equation [58,59]:

(1) $$Z_{total}=Z_{L}+Z_{H},$$

where $Z_{total}$ is the total impedance (Ωcm^−2^). $Z_{L}$ is the impedance at low frequency, and $Z_{H}$ is the impedance at high frequency.

**Figure 12:**
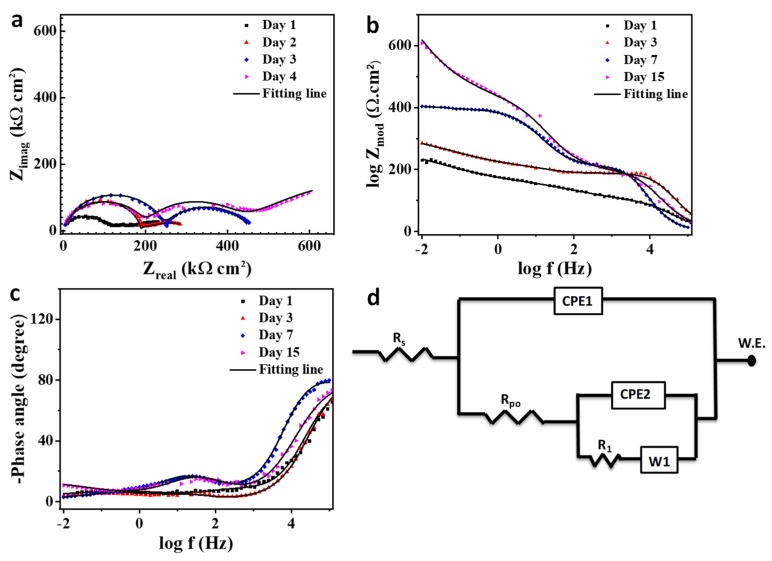
Electrochemical impedance curves for scratched plain coating immersed in 3.5 wt% NaCl for different exposure times where (**a**) Nyquist plot, (**b**) bode plot, (**c**) phase angle plot, and (**d**) the applicable equivalent circuit for the data fitting.

The electrochemical impedance graphs with the equivalent circuit for the coating modified with the self-healing microcapsule are depicted in Figure 12a–d. The R_po_ and R_ct_ shifted higher from 143.9 and 210.7 kΩ cm^2^ to 204 and 290 kΩ cm^2^, respectively, after two days of immersion. This would be due to the breakage of the microcapsules surrounding the scratched region and release of the linseed oil which has a solidification attitude on the scratched region [60]. The R_po_ and R_ct_ values continue their increase on the third day reaching to 231.9 and 401.2 kΩ cm^2^ as seen in Table 2, due to the inhibition activity of linseed oil [61]. On day 4, the R_po_ withdrew to 191.1 kΩ cm^2^ while R_ct_ reached to 550.1 kΩ cm^2^. This is probably due to the slowdown release of the linseed oil with a complete coverage for the bare steel in the scratched region which enhances the R_ct_ value [62,63]. The self-healing process is attached with a diffusion step which can be noticed in the low frequency range of impedance figures and presented as a Warburg diffusion coefficient (w) in Figure 12d. The diffusion process has a considerable effect on retarding the Cl^−^ ions penetrating through the coating as the total impedance value would follow the following equation [64,65,66]:

(2) $$Z_{total}=Z_{L}+\frac{Z_{w}}{1+j\omega Z_{w}C_{dl}}$$

(3) $$Z_{w}=Z_{H}+W$$

where *Z_w_* is the sum of the high frequency impedance and the Warburg diffusion impedance (Ω cm^−2^); *ω* is the angle frequency (rad s^−1^); *j* is an imaginary unit (−1)^1/2^; and *C_dl_* is the double layer capacitance.

The double layer capacitance is expressed by the equation [67,68]:

(4) $$C_{dl}=Y_{O}\times \omega^{n-1}$$

where *Y_O_* is the constant phase element (CPE); *ω* is the angle frequency (rad s^−1^); and n is a factor that satisfies the condition 0 ≤ *n* ≤ 1. When *n* = 1, the CPE becomes equivalent to the ideal capacitor and when *n* = 0, the CPE becomes equivalent to the resistor.

**Figure 13:**
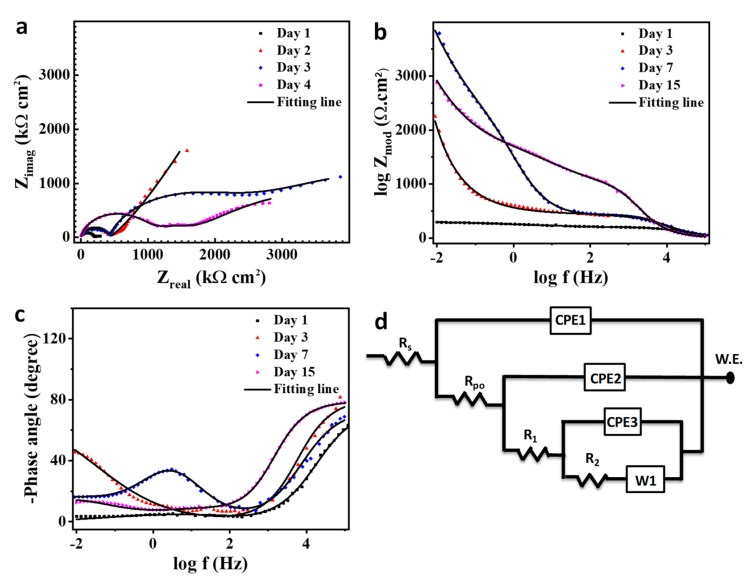
Electrochemical impedance curves for scratched smart coating immersed in 3.5 wt% NaCl for different exposure times: (**a**) Nyquist plot, (**b**) bode plot, (**c**) phase angle plot, and (**d**) the applicable equivalent circuit for the data fitting.

Figure 13 presents the impedance analysis with its equivalent circuit for the composite coating containing the self-healing microcapsule embedded with halloysite nanotubes as a carrier for NaNO_3_. The recent additives make the explanation for the mechanism of corrosion resistance ambiguous and complicated and for that reason three time constants would be used for the elucidation [69,70]. The nanocomposite coating, after one day of immersion in 3.5 wt% NaCl, gave impedance values similar to the coating with self-healing agent only, while using the best fitting data obtained from the two-time constant circuit. This is attributed to the blitz action of the self-healing process compared to the nanocontainer inhibitor release [71,72,73]. The corrosion inhibitor makes a slight change in the diffusion process as seen in Table 2. The R_po_ and R_1_ increased by 3.11 and 3.42-fold, respectively, compared to that in the previous coating. A new impedance reported (R_2_) with 3.9 MΩ cm^2^ emanated from the synergistic effect of the inhibitor release with the solidification of the linseed oil in the defected region [74]. The R_1_ and R_2_ reached to the optimum values 2.04 and 5.61 MΩ cm^2^, respectively, after three days of immersion, which agrees with the self-healing anticorrosion mechanism and giving the barrage values due to a continuous release of the inhibitor with the exposure time [19,74]. The R_po_ gives the maximum impedance after four days of immersion as a result of the complete curing of the artificial crack with the presence of the inhibitor inside the coating. It can be concluded that the sequence for the impedance magnitude for the coatings is as follows: smart coating > plain coating > pure coating.

## 4. Conclusions

Multifunctional polymeric based nanocomposite coatings were developed and their self-healing performance was evaluated. Structural and morphological analyses confirm the loading of the inhibitor and the self-healing agent into HNTs and UFMCs, respectively. The UV-Vis spectroscopic analysis indicates that self-release of the inhibitor from HNTs is sensitive to the pH of the corrosive environment and the release process itself depends upon the immersion time. The nanocomposite coatings demonstrate attractive anticorrosion properties due to the efficient release of the inhibitor and the self-healing agent from the smart containers in response to external stimuli. The improved properties of the developed nanocomposite coatings make them attractive for their potential application in the oil and gas industry.

## Figures and Tables

**Table 1:** Particle size distribution.

Size (µm)	0–4	4–63	63–125	125–250	250–500	500–1000	1000–2000
Volume (%)	0.64	9	34.96	40.88	13.07	1.32	0.13

**Table 2:** EIS parameters for neat coatings, plain coatings, and layered coatings in 3.5 wt% NaCl at room temperature.

Coating	Exposure Time, Days	Rs kΩ cm^2^	R_po_ kΩ cm^2^	Y_po_ × 10^−6^ Ω^−1^ s^n^cm^−2^	R_1_ kΩ cm^2^	Y_1_ × 10^−6^ Ω^−1^ s^n^cm^−2^	R_2_ kΩ cm^2^	Y_2_ × 10^−9^ Ω^−1^ s^n^cm^−2^	W × 10^−6^ Ω^−1^ s^n^cm^−2^
Pure	1^st^	0.916	33.17	0.814	110.3	0.094	----	----	----
	2^nd^	0.876	39.68	0.693	98.42	0.168	----	----	----
	3^rd^	0.796	46.39	0.652	81.37	0.237	----	----	----
	4^th^	0.824	36.11	0.765	73.14	0.429	----	----	----
Plain	1^st^	0.111	143.9	0.083	210.7	0.058	----	----	0.677
	2^nd^	0.241	204.7	0.059	290.0	0.046	----	----	1.34
	3^rd^	0.048	231.9	0.051	401.2	0.028	----	----	3.54
	4^th^	0.118	191.1	0.072	550.1	0.020	----	----	7.01
Smart	1^st^	0.179	128.19	0.965	216.92	0.093	----	----	1.68
	2^nd^	0.116	635.2	0.011	992.3	0.006	3916.1	6.134	0.97
	3^rd^	0.156	582.4	0.016	2041.4	0.001	5651.2	2.871	3.01
	4^th^	0.164	1213.0	0.004	1648.5	0.001	34,000	7.396	2.13
